# Mechanism of Probiotic VSL#3 Inhibiting NF-κB and TNF-α on Colitis through TLR4-NF-κB Signal Pathway

**Published:** 2019-07

**Authors:** Hui WANG, Shuhua LI, Houzhong LI, Fengxia DU, Jie GUAN, Yanmin WU

**Affiliations:** 1. Department of Immunology, Qiqihar Medical University, Qiqihar 161000, P.R. China; 2. Department of Traditional Chinese Medicine, The First Hospital of Qiqihar, Qiqihar 161000, P.R. China; 3. Department of Pharmacology, Mudanjiang Medical University, Mudanjiang 157011, P.R. China; 4. Department of Pathogen Biology, Qiqihar Medical University, Qiqihar 161000, P.R. China

**Keywords:** Probiotics VSL#3, NF-κB, TLR4, TNF-α

## Abstract

**Background:**

We aimed to investigate the effect of probiotic VSL#3 on NF-κB and TNF-α in rats with colitis and the correlation with TLR4-NF-κB signal pathway.

**Methods:**

Sixty Sprague Dawley (SD) rats were divided into the control, model and therapy groups (n=20) according to the random number table. Rats in the model and therapy groups were modeled for colitis, and rats in the therapy group were intragastrically administered with probiotic VSL#3. The expression of TLR4 and NF-κB protein, and the levels of NF-κB, TLR4, and TNF-α mRNA in the colon tissue were detected. The concentration of TNF-α in the serum after modeling but before intragastric administration (T0), 3d (T1) and 7d after intragastric administration (T2) was detected.

**Results:**

The expression of TLR4 and NF-κB p65 protein, and the levels of TLR4, NF-κB, and TNF-α mRAN in the therapy group decreased (*P* < 0.001). At T0, T1, and T2, the concentration of TNF-α in the model and control groups increased (*P* < 0.001). TLR4 and NF-κB in the therapy group were positively correlated with TNF-α mRAN (*P* < 0.050).Conclusion: In conclusion, probiotic VSL#3 inhibits the expression of NF-κB and TNF-α in rats with colitis through TLR4-NF-κB signal pathway, so it is expected to be a first choice drug for the treatment of colitis.

**Conclusion:**

In conclusion, probiotic VSL#3 inhibits the expression of NF-κB and TNF-α in rats with colitis through TLR4-NF-κB signal pathway, so it is expected to be a first choice drug for the treatment of colitis.

## Introduction

Colitis as an inflammatory lesion of the colon divided into specific and non-specific inflammations, including infectious colitis, ischemic colitis, ulcerative colitis and Crohn’s disease (1, 2), with ulcerative colitis as the most common one in clinic. With the improvement of living standards in recent years, the incidence of the disease has increased year-by-year (3).

Colitis mainly manifests as diarrhea, abdominal pain and bloody purulent stool, usually accompanied by emaciation and fatigue, and patients in severe conditions may suffer from a lesion of colon cancer (4). With unclear mechanism, the disease may be caused by bacteria, fungi, viruses and parasites (5). Due to changes and imbalance of intestinal flora and functional defects of intestinal mucosal barrier, intestinal pathogenic bacteria invade the submucosa, which activates the immune system under the mutual stimulation with microbial antigens in the body, thereby resulting in imbalance of cytokine release and inflammatory reactions (6, 7). Playing a mediatory role during the process (8), TLRs activate the translocation of NF-κB through the intracellular signal transduction of the downstream signaling molecule MyD88, thus leading to generation of TNF-α and other inflammatory cytokines (9).

Currently, colitis is mainly treated with probiotics as an adjunctive therapy, and the mechanism is to restore the balance of intestinal flora so as to reduce inflammatory cytokines and confirmatory reactions (10). As a mixture of probiotics that contains 8 common strains in the human intestinal tract, probiotic VSL#3 has been proved significantly effective in the treatment of colitis (11, 12). However, there is no research showing the exact therapeutic mechanism. Anfibatide prevents inflammatory injury induced by cerebral ischemia and reperfusion in rats through TLR4/JNK signaling pathway (13). Oridonin reduces inflammatory reactions of diabetic nephropathy by inhibiting TLR4/p38 MAPK pathway (14).

However, the effect of TLR4-NF-κB signal pathway on inflammatory cytokines has been rarely studied. Therefore, a rat model of colitis was established in this paper and the changes of NF-κB, TLR4 and TNF-α during the treatment with probiotic VSL#3 were explored, in order to prove the mechanism of action of probiotic VSL#3 in the treatment of colitis and provide clinical references.

## Materials and Methods

### Rat Information and Main Reagents

Sixty SD rats, aged 6–7 weeks and weighing 200–250 g, were separately fed in cages (5 rats for one cage). The rats were purchased from Better Biotechnology Co., Ltd., Nanjing, with an Item No. of J001; probiotic VSL#3 from Ferring Pharmaceuticals, USA; TNF-α ELISA kit from Tecan (Shanghai) Trading Co., Ltd., with an Item No. of BE45471; NF-κB Antibody from 51BioMart (Tianjin) Biotechnology Co., Ltd., with an Item No. of EL803353-100; TLR4 Antibody from Xiamen Huijia Biotechnology Co., Ltd., with an Item No. of IMG-6285A; β-Actin and horseradish peroxidase (HRP)-labeled goat anti-rat IgG (secondary antibody) from ABCAM, with Item No. of ab124964 and ab20272; fetal bovine serum (FBS), MEMα medium (low sugar), DMEM medium, Opti-MEM medium, ECL chemiluminescence detection kit, LipofectAMINE 2000 kit, RIPA and BCA protein kit from Thermo Fisher, Shanghai, China, with Item No. of 10099141, 12561072, 10569044, 11058021, 35050, 11668019, 23225 and 15224041; total RNA extraction kit EasyPure RNA Kit, PCR kit TransScript miRNA First-Strand cDNA Synthesis SuperMix and TransScript II Green Two-Step qRT-PCR SuperMix from TransGen Biotech, Beijing, China, with Item No. of ER101-01, AT351-01 and AQ301-01; 7500 PCR instrument from ABI, USA.

This experiment has been approved by the Animal Ethics Committee of our hospital.

### Methods

#### Modeling

Sixty rats were divided into three groups (n=20) according to the random number table. Rats in the control group were normally fed, whereas rats in the model and therapy groups were modeled for colitis, fasting 24 h before modeling to empty stools. The rats were modeled for colitis with reference to Yang and others (15). Before modeling, they were anesthetized with diethyl ether, and then administrated with 25 mg of 2, 4, 6-trinitrobenzenesulfonic acid (TNBS) (Sigma, with an Item No. of 107k5008) dissolved in 0.5 mL of 50% ethanol. During enema, a sterile polyethylene pipe with a diameter of 2 mm was inserted into the intestinal tract, and the anus was kept high. After enema, the rats were fed normally for 24 h. Rats in the therapy group were intragastrically administrated with probiotic VSL#3 solution daily, 0.25 mL (containing 120 mg of VSL#3 powder) each time, whereas rats in the model and control groups were intragastrically administrated with the same dose of normal saline for 7 days. All rats were sacrificed at the 8th day.

### Detection

#### Western-Blot

The colon tissue was collected from rats sacrificed to detect the expression of NF-κB p65 and TLR4 protein with Western-Blot. With the concentration detected using BCA and adjusted to 4 μg/μL, the total protein was extracted from the collected third-generation BMSCs with RIPA lysis, separated with 12% SDS-PAGE, transferred to PVDF membrane, stained with Ponceau S working solution, immersed in PBST for 5 min and washed, sealed with 5% skim milk powder for 2 h, added with primary antibody (1:1000) and sealed at 4 °C overnight. The membrane was washed to remove the primary antibody, added with secondary antibody (HRP-labeled goat anti-rat) (1:5000), incubated at 37 °C for 1 h, rinsed with PBS over 5 min each time for 3 times. The membrane was developed in a dark room, and excess liquid on the membrane was blotted with filter papers. Finally, the membrane luminesced with ECL and was developed. Protein bands were scanned to analyze the gray value using Quantity One, and the relative expression level of protein = the gray value of the target protein band / the gray value of the β-Actin protein band.

#### PCR

The colon tissue was collected from rats sacrificed to detect the expression of NF-κB, TLR4 and TNF-α mRNA with quantitative PCR (qtPCR). The EasyPure miRNA Kit was used to extract total RNA from the third-generation BMSCs, ultraviolet spectrophotometry and agarose gel electrophoresis to detect the purity, concentration and integrity. Detection of miR was as follows: TransScript® miRNA RT Enzyme Mix and 2×TS miRNA Reaction Mix were used to reversely transcribe the total RNA, with the procedure carried out in strict accordance with the manufacturer’s kit. Then, PCR amplification was carried out, and the system was as follows: 1μL of cDNA, each 0.4μL of upstream and downstream primers, 10 μL of 2×TransTaq® Tip Green qPCR SuperMix, 0.4 μL of Passive Reference Dye (50×), ddH2O used to complement to 20μL. The conditions were as follows: pre-denaturation at 94°C for 30 s, denaturation at 94 °C for 5 s, annealing and extension at 60 °C for 30 s, for 40 cycles. Three same wells were set for each sample and the experiment was performed for 3 times. In this study, β-actin was used as an internal reference, and 2^−Δct^ to analyze the data. The Primer Sequences are shown in Table 1.

**Table 1:** Primer Sequences of NF-κB, TLR4, TNF-α, and internal reference

***Primers***	***F***	***R***
NF-κB	5′-GCGCATCCAGACCAACAATAAC-3′	5′-GCCGAAGCTGCATGGACACT-3′
TLR4	5′-GCTTGAATCCCTGCATAGAG-3′	5′-ATCCAGCCACTGAAGTTCTG-3′
TNF-α	5′-GATCGGTCCCAACAAGGAGG-3′	5′-GCTTGGTGGTTTGCTACCAC-3′
β-actin	5′-TGGTGGGTATGGGTCAGAAGGACTC-3′	5′-CATGGCTGGGGTGTTGAAGGTCTCA-3′

#### ELISA

Two mL of blood was extracted from rats in the three groups after modeling but before intragastric administration (T0), 3d (T1) and 7d after intragastric administration (T2), placed at room temperature for 30min and then centrifuged for 10min (4000 rpm/min) to obtain the upper layer of the serum. ELISA was used to detect the concentration of TNF-α in the serum.

#### Observational Indexes

The expression of NF-κB p65 and TLR4 protein in the three groups of rats; the expression of NF-κB, TLR4 and TNF-α mRNA; the correlation of NF-κB and TLR4 with TNF-α mRNA in the therapy group; the concentration of TNF-α in the three groups of rats; the changes of the concentration of TNF-α in the therapy group of rats during the treatment.

#### Statistical Methods

SPSS24.0 (Shanghai Yuchuang Chemical Technology Co., Ltd.) was used to statistically calculate the results, and Graphpad8 (Softhead Inc., Shenzhen) to plot figures and check the results again. The results of this experiment were expressed as (mean±standard deviation). Repeated measures analysis of variance (ANOVA) was used for comparison between multiple time points, one-way ANOVA for comparison between groups, *t* test for pairwise comparison of normally distributed data, non-parametric test for non-normally distributed data, Pearson correlation coefficient for correlation analysis between measurement data, Spearman correlation coefficients for correlation analysis between count and measurement data. *P*<0.050 indicates a statistically significant difference.

## Results

### Results of Modeling

Three of the 40 rats for modeling died with a success rate of 92.5%. Finally, there were 20 rats modeled in the control group, 18 in the model group and 19 in the therapy group. Rats in the control group were active, and they had smooth coat color and normal eating, without diarrhea and bloody stools. Rats in the model and therapy groups had dry coat color, loose and bloody stools, with slow and small movement. However, rats in the therapy group were slightly better than those in the model group.

### Results of Western-Blot

**Fig. 1:**
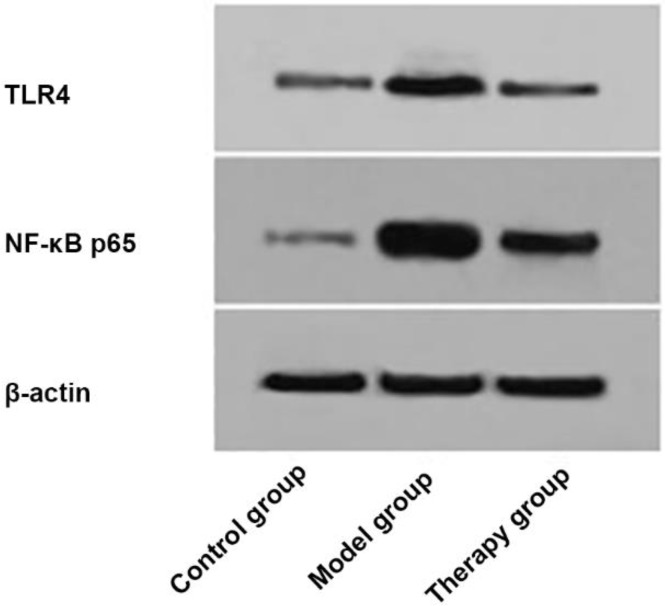
Detection results of TLR4 and NF-κB p65 protein expression The expression of TLR4 protein in the therapy group was (0.54±0.04), significantly lower than (0.82±0.07) in the model group (*P* < 0.001), but significantly higher than (0.08±0.02) in the control group (*P* < 0.001). The expression of NF-κB p65 protein in the therapy group was (0.92±0.07), significantly lower than (1.94±0.15) in the model group (*P* < 0.001), but significantly higher than (0.24±0.05) in the control group (*P* < 0.001)

The expression of TLR4 protein in the therapy group was (0.54±0.04), significantly lower than (0.82±0.07) in the model group (*P* < 0.001), but significantly higher than (0.08±0.02) in the control group (*P* < 0.001). The expression of NF-κB p65 protein in the therapy group was (0.92±0.07), significantly lower than (1.94±0.15) in the model group (*P* < 0.001), but significantly higher than (0.24±0.05) in the control group (*P* < 0.001) (Fig. 1).

### Results of PCR

**Fig. 2:**
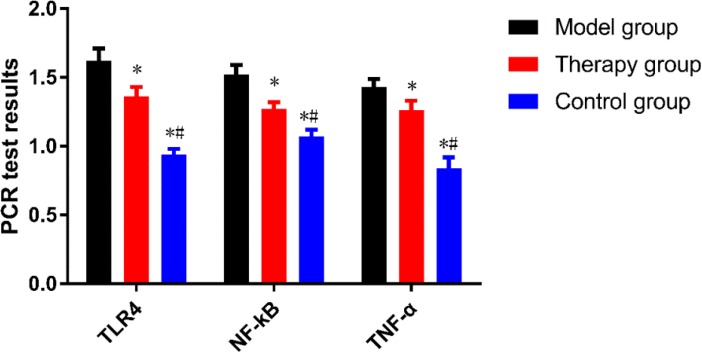
Comparison of results of PCR The levels of TLR4, NF-κB and TNF-α mRAN in the therapy group were significantly lower than those in the model group, but higher than those in the control group. * indicates *P* < 0.001 compared with the model group. # indicates *P* < 0.001 compared with the therapy group

The levels of TLR4, NF-κB and TNF-α mRAN in the therapy group were (1.36±0.07), (1.27±0.05) and (1.26±0.07), respectively, significantly lower than (1.62±0.09), (1.52±0.07) and (1.43±0.06) in the model group (*P* < 0.001), but significantly higher than (0.94±0.04), (1.07±0.05) and (0.84±0.08) in the control group (*P* < 0.001)(Fig. 2).

### Correlation of TLR4 and NF-κB with TNF-α mRAN in therapy group

**Fig. 3:**
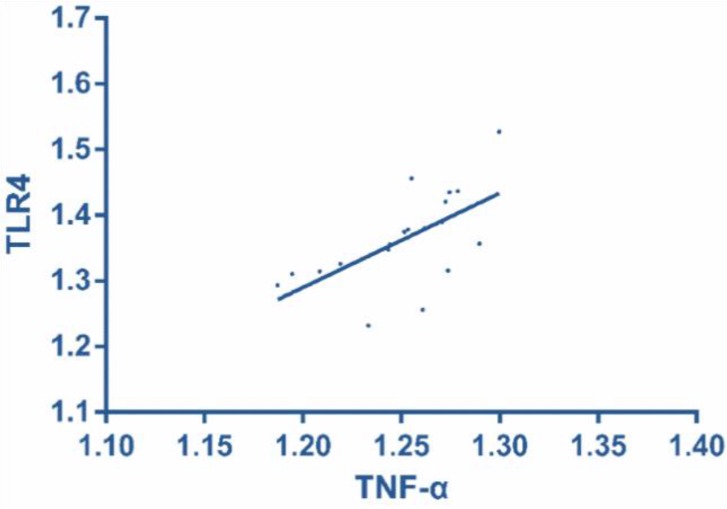
Correlation of TLR4 with TNF-α mRAN in therapy group According to Pearson correlation analysis in the therapy group, TLR4 was positively correlated with TNF-α mRAN (r = 0.619, *P* = 0.005)

**Fig. 4:**
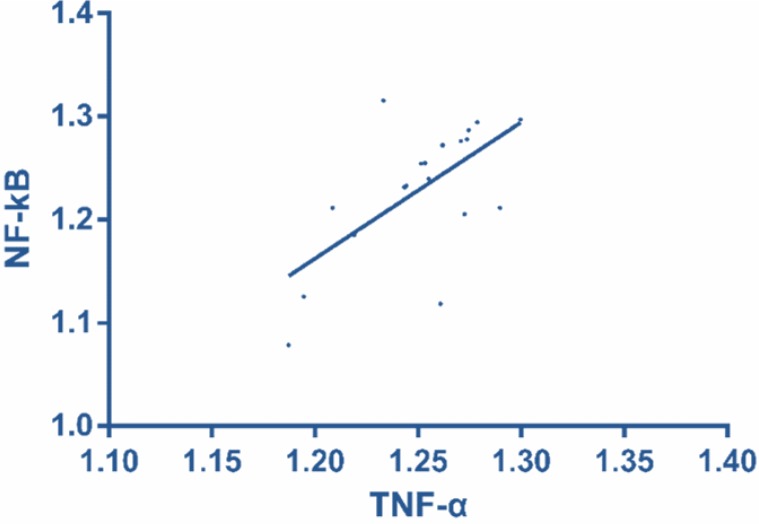
Correlation of NF-κB with TNF-α mRAN in therapy group According to Pearson correlation analysis in the therapy group, NF-κB was positively correlated with TNF-α mRAN (r = 0.629, *P* = 0.004)

According to Pearson correlation analysis in the therapy group, TLR4 was positively correlated with TNF-α mRAN (r = 0.619, *P* = 0.005), and NF-κB was positively correlated with TNF-α mRAN (r = 0.629, *P* = 0.004) (Fig. 3 and 4 and Table 2).

**Table 2:** Correlation of TLR4 and NF-κB with TNF-α mRAN in therapy group

***Variable***	***TLR4***	***NF-κB***
r	0.619	0.629
95%CI	0.229∼0.838	0.245∼0.843
R square	0.383	0.396
*P*	0.005	0.004

### Results of ELISA

**Fig. 5:**
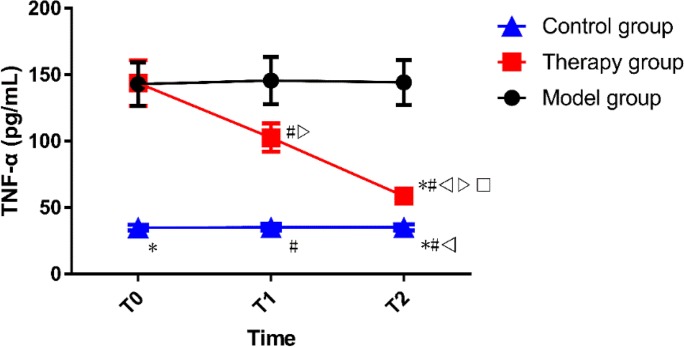
Comparison of concentration of TNF-α At T0, the concentration of TNF-α in the model and control groups was significantly higher than that in the control group. At T1 and T2, the concentration in the therapy group was significantly higher than that in the control group, but significantly lower than that in the model group. In the therapy group, the concentration at T0 was the highest, which at T1 was lower than that at T0 and which at T2 was lower than that at T1. * indicates *P* < 0.001 compared with the model group at T0. # indicates *P* < 0.001 compared with the model group at T1. △ indicates *P* < 0.001 compared with the model group at T2. ▽ indicates *P* < 0.001 compared with the therapy group at T0. □ indicates *P* < 0.001 compared with the therapy group at T1

The concentrations of TNF-α at T0, T1 and T2 were (142.85±16.33) pg/mL, (145.62±17.82) pg/mL and (144.18±16.85) pg/mL in the model group, while those were (143.72±17.15) pg/mL, (102.66±10.57) pg/mL and (58.62±3.68) pg/mL in the therapy group, and those were (34.85±2.16) pg/mL, (35.15±2.58) pg/mL and (35.08±2.38) pg/mL in the control group. At T0, there was no significant difference between the model and control groups in terms of the concentration of TNF-α, which in the two groups was significantly higher than that in the control group (*P* < 0.001). At T1 and T2, the concentration in the therapy group was significantly higher than that in the control group (*P* < 0.001), but significantly lower than that in the model group (*P* < 0.001). In the model and control groups, there was no significant difference in the concentration of TNF-α between at T0, T1 and T2. In the therapy group, the concentration at T0 was the highest, which at T1 was lower than that at T0 and which at T2 was lower than that at T1 (*P* < 0.050) (Fig. 5).

### Correlation of Concentration of TNF-α with Treatment Time

**Fig. 6:**
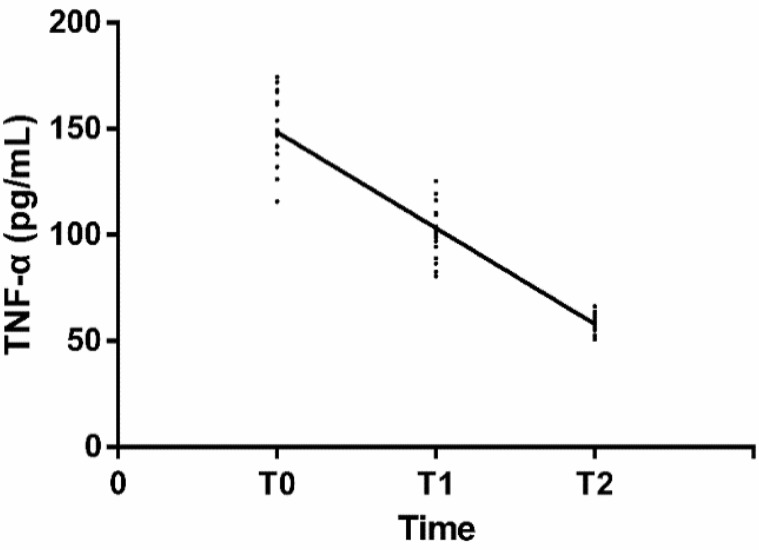
Correlation of concentration of TNF-α with treatment time According to Spearman correlation coefficients, TNF-α was negatively correlated with treatment time (r=−0.938, *P* < 0.001, 95%CI: −0.964∼−0.897)

According to Spearman correlation coefficients, TNF-α was negatively correlated with treatment time (r=−0.938, *P* < 0.001) (Fig. 6).

## Discussion

Currently, the incidence of colitis is increasing year by year (16), and the pathogenesis of it has been explored by more and more researchers at home and abroad to obtain effective prevention and treatment methods (17). Changes of bacteria in the intestinal tract and abnormalities of the immune response system are the main contents (18). Colitis is closely related to probiotics and prebiotics (19). Additionally, a study has found a significant decrease in lactobacilli and bifidobacteria in the intestinal flora of patients with colitis (20). Therefore, probiotics, which is harmless to the human body and acts on the intestinal tract, are helpful to treat colitis. VSL#3 has a high bacterial concentration, the strains in which significantly decrease in the intestinal flora of patients with colitis (21). Therefore, the influencing mechanism of VSL#3 on TNF-α was explored in this paper through its treatment of rats with colitis, in order to provide a new option for the diagnosis and treatment of patients with the disease.

In this experiment, the expression of TLR4 and NF-κB p65 protein, the levels of TLR4, NF-κB and TNF-α mRAN, and the concentration of TNF-α in the model and therapy groups were significantly higher than those in the control group, whereas the expression and the levels in the therapy group were significantly lower than those in the model group, and the concentration gradually decreased during the treatment. These findings suggest that VSL#3 can reduce the expression of TNF-α through inhibiting TLR4-NF-κB signal pathway, thereby treating colitis. TLR4 and NF-κB were closely correlated with TNF-α mRAN in the therapy group of rats, indicating that TLR4-NF-κB signal pathway is closely related to the changes of inflammatory cytokines during the treatment with probiotic VSL#3. The results of PCR were used for analysis in this study because PCR is the best method of quantitative detection and highly representative. TLR4 and NF-κB, as upstream factors of TLR4-NF-κB signal pathway, mediate and determine the ability of this pathway (22). TLR is a type I transmembrane receptor and composed of transmembrane domain, intracellular and extracellular segments. TLR4 in the TLR family is responsible for recognizing lipopolysaccharide of Gram-negative bacteria (23). As a nuclear transcription factor that is present in tissues of the human body, NF-κB is highly active and specifically binds to cells, which has been proven closely related to cell growth and inflammatory reactions in the body (24, 25). Therefore, it is speculated that the original balanced intestinal mucosal cells are stimulated by the changes of intestinal flora, which causes the intestinal flora to invade the submucosa, and activates the immune system in the body and inflammatory responses, thereby resulting in inflammatory changes in the intestinal tract and finally colitis. This process is mainly mediated by TLRs, which activates the translocation of NF-κB and causes an increase in TNF-α. The findings a study of NF-κB and TLR4 in patients with colitis are consistent with this experiment, which supports point of views in this study (26). As one of the most representative inflammatory cytokines, TNF-α is also a major pro-inflammatory factor for epithelial cell proliferation and apoptosis in the intestinal tract (27). It activates NF-κB pathway, induces the phosphorylation or ubiquitination of myosin light chain kinase, and translocates NF-κB, as well as promotes generation of inflammatory cytokines (such as IL-1β and IL-6) (28). In this study, rats in the therapy group had decreasing TNF-α during treatment, and the symptoms were improved. In the future, TNF-α targeted therapy can be used to cure colitis in clinic.

In this experiment, a rat model of colitis was established to explore probiotic VSL#3 inhibiting the expression of TNF-α in rats with colitis through TLR4-NF-κB signal pathway. However, the long-term effect of VSL#3 on colitis was not observed due to the short experimental period. Inflammatory cytokines related to colitis are not limited to TNF-α in this paper (such as IL-β and IL-6), but other cytokines were not detected and analyzed because of limited experimental conditions, so some cytokines may be more typical compared with TNF-α. There are differences between the animal and human body, so the role of VSL#3 may be deviated in the human body. In this experiment, only TNF-α was detected at multiple time points because TNF-α could be detected by ELISA during which only serum was needed. However, PCR and Western-Blot were only performed when the rats were killed because tissue detection was used for reliable experimental data. Therefore, there is no more data at multiple time points, which will be further experimented on and analyzed as soon as possible to improve the results. TNBS was used for the modeling of colitis, which is consistent with the experiments by others (29, 30). However, there are few studies on whether TNBS affects the TLR4-NF-kB signal pathway, so TNBS may increase or inhibit the expression of TLR4 and NF-kB during the modeling. Experiments on human beings will be conducted as soon as possible, and the experimental period will be expanded to improve the experiment and obtain the best experimental results.

## Conclusion

Probiotic VSL#3 inhibits the expression of NF-κB and TNF-α in rats with colitis through TLR4-NF-κB signal pathway, so it is expected to be a first choice drug for the treatment of colitis.

## Ethical considerations

Ethical issues (Including plagiarism, informed consent, misconduct, data fabrication and/or falsification, double publication and/or submission, redundancy, etc.) have been completely observed by the authors.
